# Pneumonia in Infancy and Risk for Asthma: The Role of Familial Confounding and Pneumococcal Vaccination

**DOI:** 10.1016/j.chest.2021.03.006

**Published:** 2021-03-13

**Authors:** Samuel Rhedin, Cecilia Lundholm, Emma Caffrey Osvald, Catarina Almqvist

**Affiliations:** a Department of Medical Epidemiology and Biostatistics, Karolinska Institutet, Stockholm, Sweden; b Sachs’ Children and Youth Hospital, Stockholm, Sweden; c Pediatric Allergy and Pulmonology Unit at Astrid Lindgren Children’s Hospital, Karolinska University Hospital, Stockholm, Sweden

**Keywords:** asthma, children, epidemiology, family design, pneumonia, HR, hazard ratio, ICD-10, *International Classification of Diseases, Tenth Revision*, MBR, Medical Birth Register, NPR, National Patient Register, PCV, pneumococcal conjugate vaccine, RSV, respiratory syncytial virus, SPDR, Swedish Prescribed Drug Register, TPR, Total Population Register

## Abstract

**Background:**

Studies have reported an increased risk for asthma following lower respiratory tract infections, but few studies have specifically assessed this risk in children diagnosed with pneumonia in infancy. Furthermore, it is not fully understood whether this association is indicative of a causal relationship or if certain children have a predisposition for both diseases.

**Research Question:**

Are children diagnosed with pneumonia in infancy at increased risk for asthma, and what is the role of familial confounding and pneumococcal conjugate vaccine immunization on the association?

**Study Design and Methods:**

This study was a nationwide register-based cohort analysis of > 900,000 Swedish children to assess the association between pneumonia in infancy and prevalent asthma at 4 years. A secondary aim was to assess if the association has changed after the introduction of nationwide pneumococcal conjugate vaccine (PCV) immunization as this has led to a shift in pneumonia etiology. The study controlled for important confounders, including shared environmental and familial confounding, by using sibling analyses.

**Results:**

There was a strong association between pneumonia diagnosis in infancy and prevalent asthma at 4 years (adjusted OR, 3.38; 95% CI, 3.26-3.51), as well as in the full sibling analyses (adjusted OR, 2.81; 95% CI, 2.58-3.06). The risk for asthma following pneumonia diagnosis in infancy was slightly higher for those born in the PCV period compared with the pre-PCV period (adjusted OR, 3.80 [95% CI, 3.41-4.24] vs 3.28 [95% CI, 3.15-3.42]) when the proportion of viral pneumonia etiology was also higher (14.5% vs 10.7%, respectively) and the overall asthma prevalence was lower (5.3% vs 6.6%).

**Interpretation:**

Children diagnosed with pneumonia in infancy have a highly increased risk for prevalent asthma at 4 years, which might have implications for future asthma preventive measures and needs to be considered when assessing the morbidity that can be attributed to pneumonia.

## Graphical abstract

**Figure:**
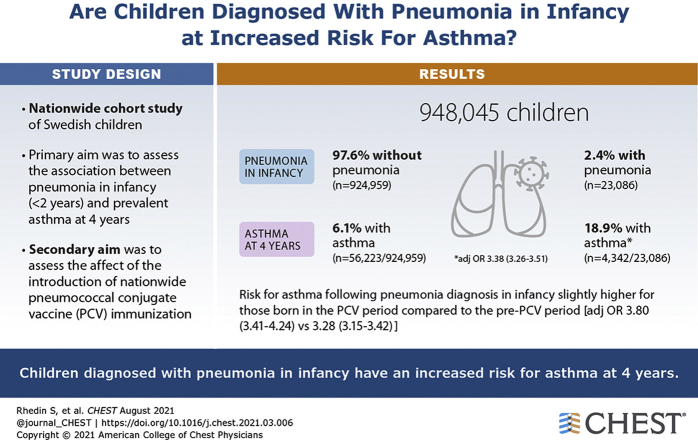

FOR EDITORIAL COMMENT, SEE PAGE 385

Asthma is a disease characterized by chronic pulmonary inflammation and bronchial hyperreactivity that contributes to significant morbidity.^1^ The etiology of the disease is multifactorial and several risk factors, both genetic and environmental, have been identified.^2^^,^^3^

Several studies have reported an association between lower respiratory tract infections and subsequent asthma, but it is not fully understood whether this association is indicative of a causal relationship or if certain children have a predisposition for both diseases.^4^, ^5^, ^6^, ^7^ Moreover, children with respiratory syncytial virus (RSV) bronchiolitis have been thoroughly studied, whereas few studies have focused on the risk for subsequent asthma in children with pneumonia.^8^, ^9^, ^10^ Pneumonia is a lower respiratory tract infection affecting the lung parenchyma, predominantly caused by the bacteria *Streptococcus pneumoniae* that contributes to significant morbidity.^11^ Historical birth cohort studies have reported an increased risk for impaired lung function in children diagnosed with pneumonia in childhood.^12^, ^13^, ^14^ However, the generalizability of these studies can be questioned given the changes in socioeconomic status, control of tobacco use, and childhood immunization that have occurred over the last decades.^15^ Furthermore, by using family design (a quasi-experimental design in which genetically related study subjects [such as siblings and half-siblings] with different exposure are compared regarding the oucome), it is possible to control for unmeasured familial confounding (ie, environmental and genetic confounding shared by the siblings or half-siblings).^16^

Introduction of pneumococcal conjugate vaccine (PCV) immunization has coincided with an overall reduction in pneumonia hospitalization rates among children and also resulted in a relative increase of viral etiology of the disease.^17^, ^18^, ^19^ In Sweden, nationwide immunization with PCV was implemented during 2007 to 2009, reaching a high coverage (> 96%) from 2011.^20^ In previous studies, viral pneumonia has been associated with the highest risk for asthma.^8^^,^^21^ Hence we hypothesized that the risk for asthma following a pneumonia diagnosis would be higher after the introduction of nationwide PCV immunization.

The primary aim of the current study was to assess the association between pneumonia diagnosis in infancy and prevalent asthma at 4 years, while controlling for important confounders, including shared genetic and familial confounding. The secondary aim was to assess if the association between pneumonia diagnosis in infancy and asthma at 4 years has changed following the introduction of the nationwide PCV immunization.

## Materials and Methods

### Study Design and Population

**Figure 1:**
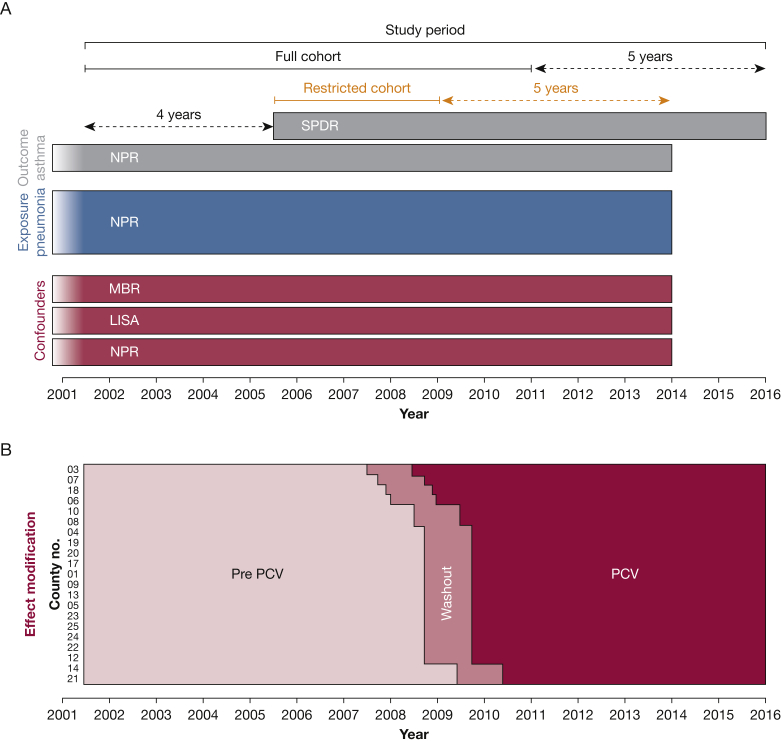
A-B, Timeline and overview of data sources. A, Overview of study cohorts and registers used. In the full cohort (children born between July 2001 and December 2010), data from SPDR were available for all children from the age of 4 years, whereas the restricted cohort used in the sensitivity analysis was limited to children with complete data from all registers for the whole study period (children born between July 2005 and December 2008). B, Overview of PCV immunization according to county. For each county, the study period was classified as occurring in the pre-PCV (the period preceding 3 months prior to the introduction date), washout (3 months prior to until 9 months following the introduction date) and PCV period (9 months following the introduction date until end of the study period). LISA = Longitudinal Integration Database for Labour Market Studies; MBR = Medical Birth Register; NPR = National Patient Register; PCV = pneumococcal conjugate vaccine; SPDR = Swedish Prescribed Drug Register.

This was a register-based cohort study. All Swedish citizens have a unique identifier, which enables linkage between the Total Population Register (TPR) and: (1) the National Patient Register (NPR), which contains *International Classification of Diseases, Tenth Revision* (ICD-10), codes for all hospital visits and approximately 80% of visits to specialist outpatient units; (2) the Swedish Prescribed Drug Register (SPDR), which was started in July 2005 and contains data on all dispensed medications; (3) the Medical Birth Register (MBR), which contains data on perinatal parameters; (4) the Longitudinal Integration Database for Labour Market Studies, containing data on education; and (5) the Multi-Generation Register, containing data on the parent’s identity of index people. Data on emigration and deaths were collected from the TPR. Register data were collected for the period July 2005 to December 2015 for SPDR and July 2001 until December 2013 for the other registers (Fig 1A). All Swedish residents born in Sweden between July 2001 and December 2010 were included (N = 965,705) to ensure that they were younger than 4 years on July 1, 2005, and older than 5 years on December 31, 2015.

**Figure 2:**
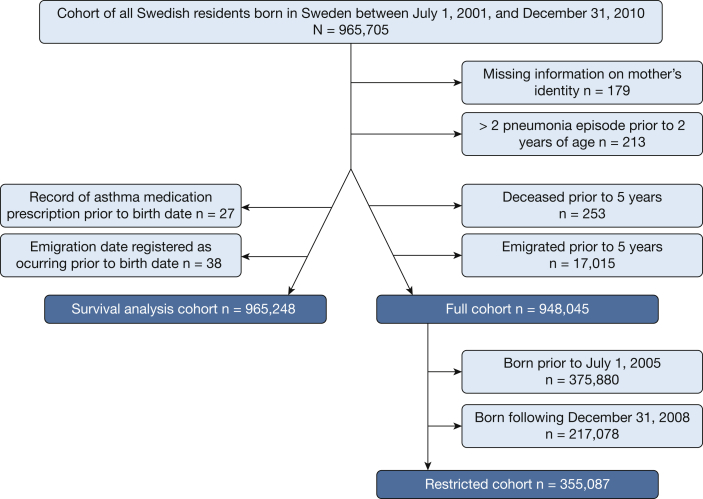
Flowchart and overview of the different cohorts used in the study.

A total of 213 children had > 2 pneumonia episodes prior to 2 years; 253 and 17,015 children, respectively, were deceased or emigrated prior to 5 years of age; and 179 children were excluded due to missing information on mother’s identity (Fig 2).

### Study Variables

The primary outcome was prevalent asthma at 4 years, which was defined according to a previously validated algorithm based on physician’s diagnosis in the NPR and dispensed asthma medications in the SPDR (e-Appendix 1).^22^^,^^23^ A secondary outcome was incident asthma/wheezing defined as fulfilling the definition for prevalent asthma at any time point during the study period.

Pneumonia in infancy was defined as a record of pneumonia (J10.0, J11.0, or J12-J18) prior to 2 years of age in the NPR. Repeated records within a time period of 8 weeks were considered to represent the same pneumonia episode, which takes into account potential routine check-ups, according to clinical practice in Sweden. Children who experienced > 2 pneumonia episodes prior to 2 years of age were excluded as these could have other underlying conditions potentially associated with asthma.^24^

Potential confounders were assessed based on qualitative assessement of the previous literature on associations between covariates and the exposure/outcome using directed acyclic graphs (e-Fig 1).^25^ Data on sex, prematurity (gestational age < 37 weeks), cesarean section, small for gestational age (< 2 SDs below expected length or weight), pregnancy type (single/multiple birth), maternal smoking during pregnancy, and parity were collected from the MBR. Data on comorbidities were collected from the NPR (e-Table 1). Data on educational level and parents’ birth country were collected from the Longitudinal Integration Database for Labour Market Studies and the TPR. Parental asthma was defined according to physician’s diagnosis and dispensed asthma medications.^22^

To assess potential effect modification from PCV immunization, the pneumonia episodes were classified as occurring in the pre-PCV period or the PCV period separated by a washout period of 3 months prior to and 9 months following the immunization starting date in each county (Fig 1B). Data on birth county were collected from the MBR.

To assess the potential impact of pneumonia etiology, the exposure was classified as bacterial/unspecified (J13-J18) or viral (J10.0, J11.0, and J12) according to ICD-10 codes. Because most viruses associated with pneumonia are almost nonexistent during summer and fall, we further classified the exposure as occurring during or outside the respiratory infections season.^17^

### Statistical Analyses

Logistic regression was used to assess the association between pneumonia in infancy and prevalent asthma, and data are presented as ORs and 95% CIs. For the adjusted estimates, two models were constructed: the first model included individual confounders (sex, prematurity, cesarean section, small for gestational age, and parity), whereas the second model also included parental factors (maternal smoking during pregnancy, educational level of parents, parents’ birth country, and parental asthma).

Etiologic fraction for asthma and 95% CIs were calculated as pneumonia prevalence in children with prevalent asthma × (adjusted OR – 1)/adjusted OR × 100.^26^

To explore confounding from familial factors, full siblings and half-siblings were identified by using the Multi-Generation Register, and all possible sibling pairs were created. Sibling comparisons were performed following exclusion of twins and multiple births using conditional logistic regression for each type of relatedness (ie, full siblings, maternal and paternal half-siblings) separately, conditioning on sibling pair, and using cluster robust SEs to account for dependence due to multiple sibling pairs within families. In the sibling analyses, adjustments were only made for individual confounders because parental factors are shared between siblings and adjusted for by design.

To assess the association between pneumonia diagnosis in infancy and incident asthma/wheezing, hazard ratios (HRs) and 95% CIs were estimated by using Cox regression analysis with pneumonia as a time-varying exposure considering pneumonia diagnoses either the first 2 years of life or any time during the study period. These analyses were performed in the survival analysis cohort also including children who emigrated or were deceased prior to 5 years but censored at death or emigration. However, 65 children were excluded due to dates for prescription of asthma medication or emigration being falsely coded as occurring before date of birth (Fig 2). Date of asthma onset was defined as the first record of either asthma medication or asthma diagnosis. Adjusted analyses (using confounders as noted earlier) were performed.

The proportional hazards assumption was tested by using Schoenfield residuals, which indicated nonproportionality (*P* < .001). Hence, we allowed for time-varying effects, assuming piecewise constant hazards 0 to 2 years and > 2 years based on graphical examination of the log hazards curves.

Five sensitivity analyses were performed (e-Appendix 1). All data analyses were conducted by using Stata version 16 (StataCorp). The Regional Ethical Review Board in Stockholm granted permission for the study.

## Results

### Description of Study Cohort

A total of 948,045 children were included in the study; of these, 23,086 (2.4%) children were diagnosed with pneumonia prior to 2 years of age. Male sex, prematurity, cesarean section, small for gestational age, maternal smoking during pregnancy, comorbidities, low parental educational level, and parental asthma were all overrepresented in children diagnosed with pneumonia compared with unexposed children (Table 1).

**Table 1:** Sociodemographic Characteristics of Study Subjects

Characteristic	All (N = 948,045)	Pneumonia Diagnosis < 2 y	
		No (n = 924,959)	Yes (n = 23,086)
Male sex	487,513 (51.3)	474,379 (51.3)	13,134 (56.9)
Prematurity (< 37 wk)	57,128 (6.0)	54,620 (5.9)	2,508 (10.9)
Cesarean section	165,839 (17.5)	160,906 (17.4)	4,933 (21.4)
Small for gestional age	20,215 (2.2)	19,441 (2.2)	774 (3.5)
Multiple birth	27,341 (2.9)	26,379 (2.9)	962 (4.2)
Maternal smoking during pregnancy	72,863 (8.1)	70,863 (8.1)	2,000 (9.2)
Comorbidity (any)	50,482 (5.3)	47,649 (5.2)	2,833 (12.3)
Chromosomal anomalies	2,486 (0.3)	2,066 (0.2)	420 (1.8)
Respiratory malformations	2,690 (0.3)	2,426 (0.3)	264 (1.1)
Cardiac malformations	18,698 (2.0)	17,471 (1.9)	1,227 (5.3)
Cerebral palsy/paralytic syndromes	2,424 (0.3)	2,182 (0.2)	242 (1.1)
Neonatal respiratory/cardiac disorders	30,139 (3.2)	28,611 (3.1)	1,528 (6.6)
Highest parental education			
Primary school (9 y)	53,307 (5.6)	51,716 (5.6)	1,591 (6.9)
Secondary school (12 y)	356,941 (37.7)	348,092 (37.7)	8,849 (38.4)
University studies (> 12 y)	492,071 (52.0)	488,588 (52.0)	11,483 (49.8)
Birth country of parents			
Mother born in Sweden	764,231 (80.6)	746,117 (80.7)	18,114 (78.5)
Mother born in other Nordic country	14,506 (1.5)	14,128 (1.5)	378 (1.6)
Mother born outside Nordic countries	169,225 (17.9)	164,635 (17.8)	4,590 (19.9)
Father born in Sweden	755,763 (80.4)	737,994 (80.5)	17,769 (77.6)
Father born in other Nordic country	15,081 (1.6)	14,685 (1.6)	396 (1.7)
Father born outside Nordic countries	169,166 (18.0)	164,446 (17.9)	4,720 (20.6)
Parity, mean ± SD	1.8 ± 1.0	1.8 ± 1.0	2.0 ± 1.1
Maternal asthma	132,047 (13.9)	128,036 (13.8)	4,011 (17.4)
Paternal asthma	104,939 (11.1)	102,000 (11.0)	2,939 (12.7)

Data are expressed as absolute number and percentage if not otherwise specified.

### Asthma Following Pneumonia Exposure

**Figure 3:**
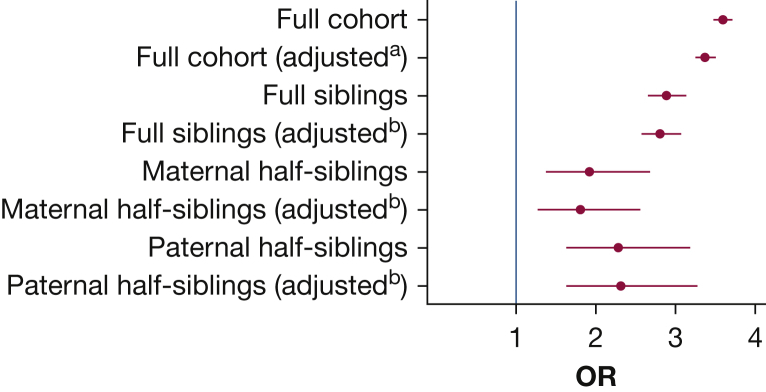
Association between pneumonia in infancy and prevalent asthma at 4 years. Point estimates expressed as ORs and 95% CIs assessed in the full cohort as well as in sibling and half-sibling analyses. ^a^Adjusted for sex, prematurity, cesarean section, small for gestational age, maternal smoking during pregnancy, parity, educational level of parents, birth country of parents, and parental asthma. ^b^Adjusted for sex, prematurity, cesarean section, and small for gestational age.

A total of 60,565 (6.4%) children had prevalent asthma at 4 years, and the cumulative incidence of asthma was 11.6% during the study period. Children diagnosed with pneumonia were more likely to have prevalent asthma at 4 years compared with unexposed children (18.9% vs 6.1%; crude OR, 3.59; 95% CI, 3.47-3.71) (e-Table 2, Fig 3). The point estimates were slightly attenuated in the adjusted models (adjusted OR, 3.38; 95% CI, 3.26-3.51). The etiologic fraction of asthma due to pneumonia diagnosis in infancy was 5.1% (95% CI, 4.7-5.4).

To adjust for genetic and shared familial confounding, sibling and half-sibling analyses were performed. After exclusion of twins, a total of 329,285 full sibling pairs, 17,312 maternal half-sibling pairs, and 16,558 paternal half-sibling pairs remained. The OR for prevalent asthma at 4 years following pneumonia diagnosis in infancy was 2.88 (95% CI, 2.65-3.14) in the full sibling analysis and 1.92 (95% CI, 1.37-2.68) and 2.28 (95% CI, 1.63-3.18) when assessing maternal and paternal half-sibling pairs, respectively (e-Table 2, Fig 3). There were only slight changes in the point estimates when additional controlling for individual confounders was performed (adjusted OR, 2.81; 95% CI, 2.58-3.06 in the full sibling analysis).

To assess incident asthma/wheezing over time, a Cox regression analysis was performed (Table 2). The HR for asthma/wheezing was higher during the first 2 years of life compared with following 2 years of age (adjusted HRs of 4.31 [95% CI, 4.11-4.51] and 1.67 [95% CI, 1.59-1.76], respectively).

**Table 2:** Cox Regression Analysis of Pneumonia Diagnosis in Infancy and Risk for Incident Asthma/Wheezing

Exposure/Outcome	Unadjusted	Model 1^a^	Model 2^b^
	HR (95% CI)	Adjusted HR (95% CI)	Adjusted HR (95% CI)
Pneumonia diagnosis < 2 y			
Asthma/wheezing 0-2 y	4.58 (4.38-4.78)	4.37 (4.18-4.57)	4.31 (4.11-4.51)
Asthma/wheezing > 2 y	1.75 (1.66-1.83)	1.68 (1.60-1.76)	1.67 (1.59-1.76)
Pneumonia diagnosis ever			
Asthma/wheezing 0-2 y	4.58 (4.38-4.78)	4.37 (4.18-4.57)	4.31 (4.11-4.51)
Asthma/wheezing > 2 y	2.28 (2.20-2.36)	2.19 (2.11-2.27)	2.18 (2.10-2.26)

HRs for incident asthma following pneumonia exposure calculated by using Cox regression. HR = hazard ratios.

a Adjusted for sex, prematurity, cesarean section, small for gestational age, and parity.

b Adjusted for sex, prematurity, cesarean section, small for gestational age, maternal smoking during pregnancy, parity, educational level of parents, birth country of parents, and parental asthma.

### Effect Modification From PCV

A total of 709,649 children were born in the pre-PCV period and 132,112 in the PCV period. Of these, 46,499 (6.6%) of the childern born in the pre-PCV period and 6,988 (5.3%) born in the PCV period fulfilled the criteria for prevalent asthma at 4 years (*P* < .001). There was significant effect modification from PCV immunization, with slightly higher OR for asthma in the PCV period compared with the pre-PCV period (adjusted ORs of 3.80 [95% CI, 3.41-4.24] vs 3.28 [95% CI, 3.15-3.42]) (Table 3).

**Table 3:** Association Between Pneumonia Diagnosis in Infancy and Prevalent Asthma in Relation to PCV Immunization and Season of Exposure

Variable	Unadjusted	Model 1^a^	Model 2^b^	*P* Value for Interaction^c^
	OR (95% CI)	Adjusted OR (95% CI)	Adjusted OR (95% CI)	
PCV immunization period				
Pre-PCV	3.50 (3.37-3.63)	3.33 (3.20-3.47)	3.28 (3.15-3.42)	.015
PCV	4.06 (3.67-4.50)	3.92 (3.53-4.35)	3.80 (3.41-4.24)	
Season of pneumonia diagnosis				
Respiratory infections season (December-April)	3.49 (3.36-3.64)	3.35 (3.22-3.49)	3.28 (3.14-3.42)	.015
Outside respiratory infections season (May-November)	3.88 (3.63-4.13)	3.65 (3.41-3.90)	3.63 (3.38-3.89)	

Association between pneumonia exposure < 2 years and prevalent asthma at 4 years in relation to PCV immunization specific for each county. PCV immunization period defined according to the introduction date of the immunization specific for each county as pre-PCV (ranging from the start of the study period until 3 months prior to the introduction), washout (ranging 3 months prior to until 9 months following the introduction), and PCV (9 months following the introduction until the end of the study period). PCV = pneumococcal conjugate vaccine.

a Adjusted for sex, prematurity, cesarean section, small for gestational age, and parity.

b Adjusted for sex, prematurity, cesarean section, small for gestational age, parity, maternal smoking during pregnancy, educational level of parents, birth country of parents, and parental asthma.

c Indicates *P* for interaction of model 2.

The majority (88.7%) of pneumonia episodes were classified as bacterial/unspecified and 11.3% as viral. The proportion of viral etiology was significantly higher in the PCV period compared with the pre-PCV period (14.5% vs 10.7%, respectively; *P* < .001). There were minor differences in ORs for asthma comparing children diagnosed with pneumonia during or outside the respiratory infections season (Table 3).

### Sensitivity Analyses

The first four sensitivity analyses did not result in any major changes of the point estimates (e-Table 3). In the fifth sensitivty analysis, the association of pneumonia diagnosis in infancy and the risk for asthma was assessed following exclusion of all children with a record of any dispensed asthma medication or asthma diagnosis before 2 years of age. This resulted in a markedly decreased asthma prevalence (3.4%) and clearly attenuated but still significant point estimates (adjusted OR, 1.66; 95% CI, 1.48-1.87).

## Discussion

In this large-scale register-based cohort study, we report a strong association between pneumonia diagnosis in infancy and prevalent asthma at 4 years of age and that the strength of the association has increased slightly after the introduction of PCV immunization, possibly related to a relative increase in viral pneumonia etiology. Children diagnosed with pneumonia in infancy had clearly increased odds for asthma even following adjustments for important confounders, including shared genetic and familial environment assessed by using sibling analyses.

Our findings are in line with a study by Chan et al,^21^ who reported an increased risk for asthma (OR, 1.95; 95% CI, 1.11-3.44) assessed by spirometry at 11 to 26 years of age following pneumonia exposure in infancy in an American birth cohort study of > 1,000 children born 1980 to 1984. We expand this further by also adjusting for familial confounding and addressing potential effect modification from PCV immunization. Their lower point estimates could potentially be explained by a longer follow-up period and a more strict definition of asthma based on spirometry at 11 to 22 years of age, as some children outgrow their asthma during adolescence but could also be a result of loss of follow-up or differences in etiology. Other birth cohort studies have reported a strong long-term risk for predominantly restrictive deficits.^13^^,^^14^ Several studies have suggested that viral pneumonia is more strongly associated with asthma than bacterial pneumonia.^8^^,^^21^ This is further supported by a Dutch randomized controlled study by Blanken et al^4^ showing that RSV prophylaxis with palivizumab is associated with a reduction in recurrent wheezing and parental reported asthma; howeveer, the follow-up study did not reach the primary outcome of a reduction in physician-diagnosed asthma at 6 years of age.^10^ There are limited data on the assocation between pneumonia and asthma in low-income countries. Data from a South African birth cohort suggest that lower respiratory tract infections in infancy are associated with decreased lung function and recurrent wheeze.^27^^,^^28^

Several different biological mechanisms could explain the association between pneumonia diagnosis and asthma, including direct damage to the lung parenchyma, immunomodulatory mechanisms, or disturbance of lung development during a critical phase. In mouse models, pneumococcal pneumonia has been shown to alter the expression of airway smooth muscle proteins, whereas RSV infection has been associated with immune modulation and airway hyperresponsiveness.^29^^,^^30^

It has been argued that some children are genetically predisposed for both severe respiratory tract infections and asthma, and it has not been clear whether there is a causal association between the respiratory infection and development of asthma.^31^ By using a quasi-experimental design with sibling controls, we were able to further expand the previous knowledge on the association between pneumonia and asthma.^16^ Briefly, the rational for the sibling analyses is to control for unmeasured familial confounding (ie, environmental or genetic factors shared by the siblings or half-siblings). If the point estimates in the sibling analysis are attenuated compared with those in the standard observational analyses, it suggests that the association is confounded by familial factors. In contrast, if the effect remains in the sibling analyses, it does not prove, but provides additional support for, a causal link between the studied exposure and the outcome, as compared with ordinary analyses of observational studies.^16^ In our study, the point estimates in the sibling analyses were only slightly attenuated compared with the ordinary analyses, which points toward a causal link between the pneumonia diagnosis in infancy and the prevalent asthma assessed at 4 years of age.

We assessed effect modification from PCV immunization, as nationwide PCV immunization of children was introduced during the study period, which has likely changed the etiology of pediatric pneumonia.^19^ Children born in the PCV period had a slightly increased risk for asthma if diagnosed with pneumonia in infancy compared with those born in the pre-PCV period. This supports the hypothesis that viral pneumonia is more strongly associated with asthma and that the absolute risk for asthma in children experiencing pneumonia in infancy is somewhat higher today, potentially owing to the relative increase in viral etiology of childhood pneumonia.^8^^,^^21^ Further supporting this hypothesis was an observed relative increase in viral pneumonia diagnosis codes in the PCV period compared with the pre-PCV period and higher point estimates when restricting the exposure definition to only include viral pneumonia diagnosis codes in a sensitivity analysis. However, specific ICD-10 codes should be interpreted cautiously as pneumonia etiology is hard to establish in children, and coding practices might change over time.^32^^,^^33^ As an alternative approach, we compared children diagnosed with pneumonia during and outside the respiratory infections season but observed only minor differences in the point estimates. Of note, we observed an overall lower asthma prevalence in children born in the PCV period compared with those born in the pre-PCV period. This raises questions about a potential asthma-reducing effect of PCV, although this topic is beyond the scope of the current study.

Because asthma itself is a risk factor for severe pneumonia, there is a risk of potential reverse causation (ie, the asthma disease was already present at the time for exposure and thus increased the risk for acquiring pneumonia). However, in a survival analysis where we allowed for both time-varying exposure and time-varying effect, there was still a clear association between pneumonia and incident asthma/wheezing. Nevertheless, the association was weaker following 2 years of age compared with during the first 2 years. This finding is likely partly explained by the fact that the majority of wheezing episodes prior to 2 years of age represents viral-induced wheeze rather than true asthma.^34^ We also performed a sensitivity analysis, in which all children with an asthma diagnosis or any dispensed asthma medication prior to 2 years of age were excluded to ascertain that the pneumonia preceded the asthma diagnosis. In this analysis, the point estimates were clearly attenuated, but the association was still present. Together, this points toward an immediate increased risk for asthma following pneumonia exposure in the youngest children, coinciding with a critical phase of the lung development and maturation of the immune system and potentially representing an infectious asthma phenotype that, to a higher degree, can be outgrown. We cannot, however, completely rule out reverse causation (ie, the increased risk for asthma seen in children diagnosed with pneumonia in infancy was partly explained by undiagnosed asthma at the time of exposure). There are also studies suggesting that respiratory infections might trigger asthma development in the presence of certain genetic variants, further complicating the interpretation of the direction of the association.^35^

A strength of the study was the large study population covering the entire Swedish population including > 900 000 children, the nearly complete follow-up data owing to high-quality health and population registers held in Sweden, previously validated definitions of asthma, and our family-based approach specifically assessing environmental and genetic confounding shared between siblings.^16^ This allowed proper controlling for several important confounders, as well as assessment of effect modification from PCV immunization.

There are also some limitations of the study. First, pneumonia in children is mainly diagnosed clinically, and the disease presentation of pneumonia, other lower respiratory tract infections, and asthma overlaps.^36^ To account for this, some epidemiologic pneumonia studies have used a radiology-based case definition favoring specificity over sensitivity, whereas we assessed physician-diagnosed pneumonia according to ICD-10 codes.^37^ According to clinical practise in Sweden, a large proportion (although not all) of infants with pneumonia have a radiology-based diagnosis. Using a strict radiographic pneumonia definition provides a more robust but less sensitive definition, which affects the generalizability as a large number of children are clinically diagnosed with pneumonia. It is possible, however, that in our study, some wheezing episodes early in life were misclassified as pneumonia, potentially overestimating the association.^38^ Second, we could not include visits to general practitioners, as they are not recorded in the NPR. However, there are national guidelines from the Swedish Public Health Agency and the Medical Products Agency recommending that children with suspected pneumonia should be referred to a pediatric clinic if they are < 6 months of age or if they present with respiratory distress (age-adjusted tachypnea, grunting, severe indrawings, or peripheral oxygen saturation < 93%).^39^ Hence, the vast majority of pneumonia episodes should be included. Third, the diagnosis of asthma in young children is complex and represents a heterogenous group of wheezing phenotypes.^34^^,^^40^ We assessed prevalent asthma at 4 years of age as our main outcome, using a previously validated algorithm based on both physician’s diagnosis and dispensed asthma medication requiring evidence of an active disease during the fourth year of life and also performed several different sensitivity analyses that did not have any major effects on the point estimates.^22^ Future studies should assess the long-term risk for asthma following pneumonia diagnosis, target beneficial effects from pneumonia interventions (including PCV immunization), and explore the association between pneumonia and asthma in low-income countries, where currently limited data are available but where future interventions would likely have the most impact.

## Interpretation

We report that pneumonia diagnosis in infancy is strongly associated with prevalent asthma at 4 years despite controlling for important confounders, including genetic and shared familial environment. If this association is causal, it implies further beneficial effects from interventions targeting pneumonia.

**Take-home Points**

**Study Question:** Is pneumonia diagnosis in infancy associated with subsequent asthma, and what is the role of familial confounding and PCV immunization on the association?

**Results:** We report a strong association between pneumonia diagnosis in infancy and prevalent asthma at 4 years of age in this nationwide register-based cohort study of > 900,000 Swedish children despite thorough controlling for important confounders. We also found that the strength of the association has increased slightly since the introduction of PCV immunization, possibly related to a relative increase in viral pneumonia etiology.

**Interpretation:** The findings might have implications for future pneumonia preventive measures and need to be considered when assessing the morbidity that can be attributed to pneumonia.

## Supplementary Data

e-Online Data
