# Supplementary material for: Pneumonia in Infancy and Risk for Asthma: The Role of Familial Confounding and Pneumococcal Vaccination
Source: Chest. 2021 Mar 13;160(2):422–31. doi: 10.1016/j.chest.2021.03.006 (PMC8411448; doi:10.1016/j.chest.2021.03.006)
Supplement: e-Online Data [file mmc1.pdf]

CHEST Online Supplement

Pneumonia in Infancy and Risk for Asthma

The Role of Familial Confounding and Pneumococcal Vaccination

Samuel Rhedin, PhD; Cecilia Lundholm, MSc; Emma Caffrey Osvald, MD; and Catarina Almqvist, MD, PhD

CHEST 2021; 160(2):422-431

Online supplements are not copyedited prior to posting and the author(s) take full responsibility for the accuracy of all data.

© 2021 AMERICAN COLLEGE OF CHEST PHYSICIANS. Reproduction of this article is prohibited without written permission from the American College of Chest Physicians. See online for more details. **DOI:** 10.1016/j.chest.2021.03.006

CHEST Online Supplement

e-Appendix 1.

Asthma definition

Prevalent asthma at 4 years was defined as: I) a) having record of asthma (ICD-10 code J45) in the NPR and either b) having ≥2 records of dispensed inhaled corticosteroids (R03BA), β2-agonists/corticosteroids combination drugs (R03AK06 or R03AK07) or leukotriene receptor antagonists (R03DC03) independent of time between distributions or c) having ≥3 records of any medication listed above or inhaled β2-receptor agonist (R03AC02, R03AC03, R03AC12 or R03AC13), dispensed within a 12-month period AND II) having a record of asthma (J45) in the NPR or a record of dispensed asthma medication (any of those listed above) in the SPDR during the fifth year of life.[20,21] Incident asthma/wheezing was defined as meeting the criteria for I) but not II). In a sensitivity analysis, we assessed an alternative less strict definition of prevalent asthma, which allowed for a record of asthma diagnosis or dispensed asthma medication in an expanded time period of +/- 9 months at 4.5 years. In this sensitivity analysis, the study cohort was limited to children born October 1st 2001 – September 31st 2010 to have complete SPDR data of all study subjects 9 months before and after they turned 4.5 years.

Sensitivity analyses

Five sensitivity analyses were performed. First the exposure was restricted to either bacterial/unspecified or viral pneumonia ICD-10 codes. Second, an alternative less strict outcome definition of prevalent asthma was used (see definition above). Third, the association between pneumonia diagnosis in infancy and prevalent asthma was assessed after exclusion of children with comorbidities to reduce potential confounding and misclassification bias as certain lung malformations and cardiac wheeze are treated similarly as asthma. Fourth, analyses were performed in the restricted cohort, where complete data from all registers used was available. Fifth, the analyses were performed in the restricted study cohort after exclusion of children with asthma diagnosis or dispenses of asthma medications during the first 2 years of life to address reverse causality and so ensures that the pneumonia diagnosis occurred prior to the outcome of asthma.

Online supplements are not copyedited prior to posting and the author(s) take full responsibility for the accuracy of all data.

e-Table 1 – List of ICD-10 codes for comorbidity categories

Cardiac malformations

Q20 Congenital malformations of cardiac chambers and connections
Q21 Congenital malformations of cardiac septa
Q22 Congenital malformations of pulmonary and tricuspid valves
Q23 Congenital malformations of aortic and mitral valves
Q24 Other congenital malformations of heart
Q25 Congenital malformations of great arteries
Q26 Congenital malformations of great veins

Respiratory malformations

Q30 Congenital malformations of nose
Q31 Congenital malformations of larynx
Q32 Congenital malformations of trachea and bronchus
Q33 Congenital malformations of lung
Q34 Other congenital malformations of respiratory system

Chromosomal anomalies

Q90 Down syndrome
Q91 Trisomy 18 and Trisomy 13
Q92 Other trisomies and partial trisomies of the autosomes, not elsewhere classified
Q93 Monosomies and deletions from the autosomes, not elsewhere classified
Q95 Balanced rearrangements and structural markers, not elsewhere classified
Q96 Turner's syndrome
Q97 Other sex chromosome abnormalities, female phenotype, not elsewhere classified
Q98 Other sex chromosome abnormalities, male phenotype, not elsewhere classified
Q99 Other chromosome abnormalities, not elsewhere classified

Cerebral palsy and paralytic cyndromes

G80 Cerebral palsy
G81 Hemiplegia and hemiparesis
G82 Paraplegia (paraparesis) and quadriplegia (quadriparesis)
G83 Other paralytic syndromes

Neonatal respiratory and cardiac disorders

P19 Metabolic acidemia in newborn
P20 Intrauterine hypoxia
P21 Birth asphyxia
P22 Respiratory distress of newborn
P23 Congenital pneumonia
P24 Neonatal aspiration
P25 Interstitial emphysema and related conditions originating in the perinatal period
P26 Pulmonary hemorrhage originating in the perinatal period
P27 Chronic respiratory disease originating in the perinatal period
P28 Other respiratory conditions originating in the perinatal period
P29 Cardiovascular disorders originating in the perinatal period

Online supplements are not copyedited prior to posting and the author(s) take full responsibility for the accuracy of all data.

e-Table 2 – Association between pneumonia in infancy and prevalent asthma

	**Asthma at 4 years**			
	n/N (%)	Unadjusted OR (95% CI)	Model 1[a] OR (95% CI)	Model 2[b] OR (95% CI)
**Full cohort**				
Pneumonia diagnosis <2 years				
No	56 213/924 959 (6.1)	Ref	ref	ref
Yes	4 352/23 086 (18.9)	3.59 (3.47-3.71)	3.43 (3.31-3.55)	3.38 (3.26-3.51)
**Family analyses**				
Full siblings (n=329 285 total full sibling pairs; n=3 330 discordant pairs)				
Pneumonia diagnosis <2 years				
No		ref	ref	NA
Yes		2.88 (2.65-3.14)	2.81 (2.58-3.06)	NA
Maternal half-siblings (n=17 312 total maternal half-sibling pairs; n=175 discordant pairs)				
Pneumonia diagnosis <2 years				
No		ref	Ref	NA
Yes		1.92 (1.37-2.68)	1.80 (1.27-2.56)	NA
Paternal half-siblings (n=16 558 total paternal half-sibling pairs; n=205 discordant pairs)				
Pneumonia diagnosis <2 years				
No		ref	Ref	NA
Yes		2.28 (1.63-3.18)	2.31 (1.63-3.27)	NA

[a]Adjusted for sex, prematurity, cesarean section, small for gestational age and parity.
[b]Adjusted for sex, prematurity, cesarean section, small for gestational age, maternal smoking during pregnancy, parity, educational level of parents, birth country of parents and parental asthma. Abbreviations: CI, confidence interval; OR, odds ratio.

Online supplements are not copyedited prior to posting and the author(s) take full responsibility for the accuracy of all data.

e-Table 3 – Sensitivity analyses

	Asthma at 4 years			
	n/N (%)	Unadjusted OR (95% CI)	Model 1[a] OR (95% CI)	Model 2[b] OR (95% CI)
**I) Alternative exposure definition (restricting to viral or bacterial/unspecified pneumonia ICD-10 codes)**				
a) Bacterial/unspecified pneumonia (ICD-10: J13-J18, A39.1)				
Pneumonia diagnosis <2 years				
No	56 903/928 352 (6.1)	ref	ref	ref
Yes	3 662/19 693 (18.6)	3.50 (3.37-3.63)	3.35 (3.22-3.48)	3.29 (3.16-3.43)
b) Viral pneumonia (ICD-10: J10.0, J11.0, J12)				
Pneumonia diagnosis <2 years				
No	59 696/944 129 (6.3)	ref	ref	ref
Yes	869/3 916 (22.2)	4.23 (3.92-4.56)	4.00 (3.69-4.33)	3.93 (3.61-4.27)
**II) Alternative outcome defintion (expanded time-period to 4.5 years +/- 9 months)**				
Pneumonia diagnosis <2 years				
No	63 956/924 959 (6.9)	ref	ref	ref
Yes	4 935/23 086 (21.4)	3.66 (3.54-3.78)	3.51 (3.39-3.63)	3.46 (3.34-3.59)
**III) Children with comorbidities excluded**				
Pneumonia diagnosis <2 years				
No	51 252/877 310 (5.8)	ref	ref	ref
Yes	3 490/20 253 (17.2)	3.36 (3.23-3.48)	3.29 (3.17-3.42)	3.21 (3.08-3.34)
**IV) Restricted cohort**				
Pneumonia diagnosis <2 years				
No	23 066/345 943 (6.7)	ref	ref	ref
Yes	1 765/9 153 (19.3)	3.34 (3.17-3.53)	3.20 (3.03-3.39)	3.13 (2.96-3.23)
**V) - Restricted cohort. children with asthma diagnosis/medication before 2 years excluded**				
Pneumonia diagnosis <2 years				
No	10 837/315 690 (3.4)	ref	ref	ref
Yes	343/6 014 (5.7)	1.70 (1.52-1.90)	1.70 (1.52-1.90)	1.71 (1.52-1.92)

[a]Adjusted for sex, prematurity, cesarean section, small for gestational age and parity.
[b]Adjusted for sex, prematurity, cesarean section, small for gestational age, maternal smoking during pregnancy, parity, educational level of parents, origin of parents, parental asthma.
Abbreviations: CI, confidence interval; OR, odds ratio.

Online supplements are not copyedited prior to posting and the author(s) take full responsibility for the accuracy of all data.

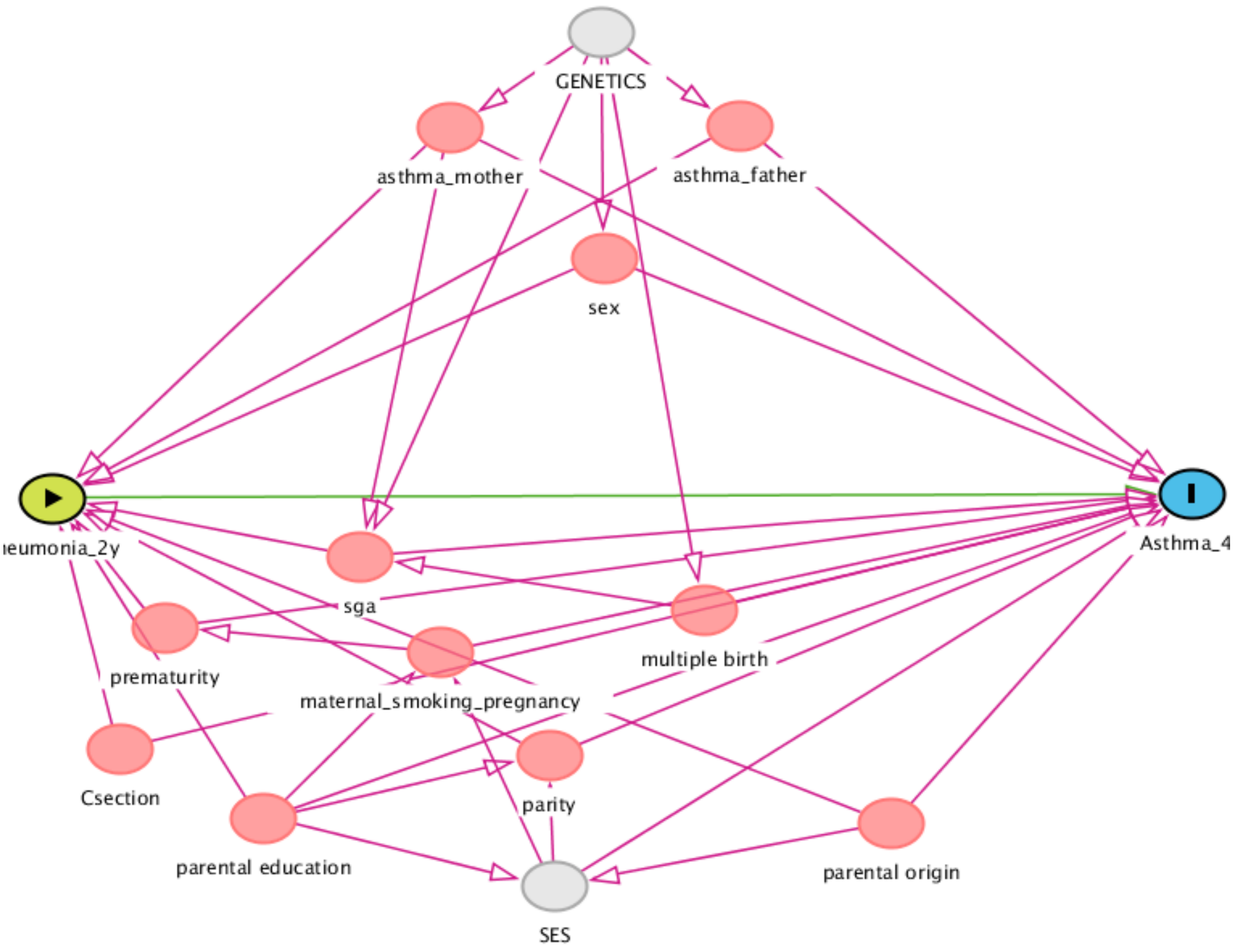

e-Figure 1. Identified confounders in study of pneumonia diagnosis in infancy and prevalent asthma at 4 years. Directed acyclic graph of potential confounders (pink) to the association between pneumonia diagnosis (green) in infancy and outcome asthma at 4 years (blue). Abbreviations: Csection, cesarean section; SES. Socioeconomic status; SGA, small for gestational age.

205695

Online supplements are not copyedited prior to posting and the author(s) take full responsibility for the accuracy of all data.
